# Descriptive study for culling and mortality in five high-producing Spanish dairy cattle farms (2006–2016)

**DOI:** 10.1186/s13028-018-0399-z

**Published:** 2018-07-28

**Authors:** Ramon Armengol, Lorenzo Fraile

**Affiliations:** 1 Departament de Ciència Animal, ETSEA, University of Lleida, Av. Alcalde Rovira Roure 191, E25198 Lleida, Spain; 2 Agrotecnio Center, Av. Alcalde Rovira Roure, 80, E25198 Lleida, Spain; 3 Present Address: Department of Animal Science, University of Lleida, ETSEA, Av. Alcalde Rovira Roure, 191, E25198 Lleida, Spain

**Keywords:** Benchmarking, Dairy cow, Culling, Mortality

## Abstract

**Background:**

High turnover rate in dairy farms due to culling and mortality is associated with poor animal welfare, an increase in production costs and lower economic benefits for the dairy farm. Understanding cow elimination better would help to formulate specific prevention measures and improve the efficiency of milk production in dairy farms. Culling and mortality data from five standard high-producing dairy farms in Spain were analysed over a period of 11 years (2006–2016). Data were collected by the same veterinary team and using the same software system.

**Results:**

Significant between-herd differences in eliminated cows were observed for type of elimination (slaughter or death on the farm), age at elimination, cause of elimination, number of lactations and production parameters, such as total days in milk, life milk yield, litres per day of productive life and litres per day of life. Culling and mortality were higher during the hot season and for cows of second and third parities. Between-herd differences were observed. Reproductive disorders (30.2%) were the most frequent reason for elimination, with low production (23.4%) being the second most frequent reason. Accidents on the farm (7.7%) were a more frequent cause of elimination than metabolic diseases (7.2%), locomotor disorders (2.4%) and obstetrics (2.4%).

**Conclusions:**

Veterinary teams or farmers’ associations can use culling and mortality information for benchmarking cow farms if data collection and analysis is standardised for comparability. The analysis of culling and mortality information should help farmers to improve efficiency.

**Electronic supplementary material:**

The online version of this article (10.1186/s13028-018-0399-z) contains supplementary material, which is available to authorized users.

## Background

Culling (removal of a live cow from the farm for slaughter), mortality (death of an animal on the farm, whether euthanized or unassisted) and sale (selling cows to other farms) are normal events in intensive dairy production systems. In any livestock production system, net farm revenue is affected by income and costs. The sale of milk is the most important factor for income in dairy farms and the rearing and purchase of replacements are important costs for the farm [1].

A high turnover rate negatively affects the farm´s average milk production due to the fact that replacements produce less milk than the substituted animals in the short term [2, 3]. Moreover, a high turnover rate directly increases the cost of replacement at the farm level. Thus, a thorough analysis of the causes of elimination and death of these cows is imperative to improving productive efficiency. It is also increasingly a concern for dairy product consumers and animal welfare, because a high and sustained turnover rate is an indicator of poor welfare status [4]. Ideally, farmers should voluntarily decide which cows go to slaughter based on production and should be able to replace these cows with first lactation cows with a higher potential of production.

Spain and the southern part of Europe have a long heat stress risk season (May–October) that can affect the reproductive performance and immunologic status of cows [5]. Moreover, the prevalence of metabolic disorders, mastitis and infectious diseases and/or their severity can also be significantly affected by climate [6]. Thus, weather conditions could affect culling and mortality parameters throughout the year.

The causes of culling and mortality in dairy cows are associated with reproductive status and disorders [7–9], milk production [10], metabolic and post-partum disorders, mammary gland disorders and lameness [11–13]. A portion of culling and mortality events on dairy farms can be attributed to undesirable factors in the production system. These causes vary depending on age, parity and stage of lactation of the dairy cow [12, 14]. In some areas of the world, the proportion of dairy cows eliminated by a particular cause may vary throughout the year according to extrinsic factors on the farm, such as climate [15]. Moreover, each farm has its own elimination policy for dairy cows and the percentage of animals involuntarily culled or dying on the farm is specific to each farm. Studying the causes and percentages of elimination and mortality at different times of the year can help identify seasonal problems in the production system. This type of analysis can also inform future investment decisions to improve animal welfare and production. Using the same criteria and analysis system is necessary to advice farmers and benchmark different farms. The main aim of the study was to describe the causes of culling and mortality on selected high-producing dairy farms and establish benchmarking indicators.

## Methods

### Study population

Five standard high-production commercial dairy herds from North-eastern Spain (Huesca in Aragon and Lleida in Catalonia), of the Holstein Fresian breed, were selected from the database of a veterinary practice. Selection criteria for including a farm were: use of the same farm software, same codification for culling and mortality reasons, similar management systems and the same veterinary team carrying out clinical and/or anatomo-pathological examination for diagnosing the causes of culling and mortality. A high-producing dairy farm with Holstein cows produces at least 10,000 l of milk by cow in 305 days of lactation [16]. The study was carried out on five high-production commercial dairy farms over a period of 11 years (1st January 2006 to 31st December 2016). Farms housed between 140 and 600 lactating Holstein cows (bovine females that had calved at least once) with a production of 10,300–12,300 kg of milk by cow (3.6% fat and 3.3% protein) in 305 days. Cows were housed in sawdust-bedded free-stall barns or sawdust-bedded free-stall cubicles, and were fed a total mixed ration consisting of corn silage, grass silage, and concentrates and cows were never grazed. Cows were milked twice (at 6 a.m. and 6 p.m.) or three times a day (at 4 a.m., 12 p.m., and 8 p.m.) and individual milk yield was recorded daily. Each lactating cow was sampled and analysed by the Official Milk Recording Centre for milk quality (fat, protein and lactose concentration) and somatic cell count per ml once a month. Because of data ownership issues, official milk quality parameters were not available for this study. Herd size of the farms under study was stable throughout the period (± 3%). Breeding management was carried out by artificial insemination with Holstein semen. Cows were bred on observed oestrus or diagnosed by a computerised pedometry system (Afimilk, Kibbutz Afikim, 1514800, Israel). It was decided not to inseminate first lactation and multiparous cows before 90 and 70 days postpartum, respectively.

For the purpose of this study, the year was divided into four periods as detailed in Table 1. Finally, farms 1, 3 and 4 had cooling systems based on the use of fans and showers, farm 2 used showers and farm 5 had no cooling system.

**Table 1:** Description of the characteristics of the different periods of the year

Period	1	2	3	4
Months	1st January–31st March	1st April–30th June	1st July–30th September	1st October–31st December
Avg maximum temp (°C)	12.7	25.1	31.1	15.6
Avg minimum temp (°C)	0.8	14.3	17.4	5.2
Avg relative humidity (%)	71	55	57	79

*Avg* average, *Temp* temperature

### Destiny and cause of elimination

The destiny of eliminated cows was classified into two groups: slaughtered or dead on the farm. The average annual herd turnover rate was defined as the mean number of eliminated cows per year over the 11-year period divided by the mean number of cows present during the 11-year period [17]. In the included farms, the sale of animals for further productive purposes did never occur. The cause of elimination was established based on the classification proposed by the USDA in the National Animal Health Monitoring Scheme [1, 18]. The cause of elimination was established consensually between the veterinarian and the farmer, taking into account the veterinary diagnosis and the productive, genetic improvement and milk quality criteria of the farmer. Although the software is able to capture different possible causes of elimination, the database only retains the primary cause of elimination in the cow’s individual history. The causes of elimination are detailed below.

#### Reproductive causes

Cows with reproductive pathology such as abortion, ovarian cysts, anovulatory cows, non-pregnant first lactation and multiparous cows up to 350 and 250 days in milk (DIM), respectively. Non-pregnant cows remained on the farm until their average daily milk production was under 22 l/day.

#### Mammary gland causes

Mastitis (clinical, subclinical or chronic) of any of the quarters, cistern or teat morphological defects.

#### Production causes

Cows that are not producing enough milk within 40–200 DIM according to the prediction of milk yield provided by the farm software (approximately 22 l/day over 7 consecutive days) without an overt clinical problem and without taking into account their pregnancy status.

#### Locomotor causes

Limping cows and morphological leg defects.

#### Metabolic/digestive causes

Abomasal torsion, dilation or displacement, diarrhoea, ketosis, fatty liver, hypocalcaemia, hypomagnesaemia, hypokalaemia, acidosis and downer cow syndrome.

#### Respiratory causes

Bovine respiratory syndrome confirmed by clinical or necropsy examination.

#### Dystocia/obstetrics causes

Clinical problems related to calving such as dystocia, uterine torsion, prolapsed uterus or vagina. Uterine, vaginal or vulvar trauma as a consequence of parturition were also included. Nervous and musculoskeletal system lesions as a consequence of dystocia were included if they occurred within the first 7 days after parturition.

#### Accidental causes

Trauma with equipment, tools or buildings on the farm. Errors in drug administration, intoxication with chemicals and traumatic reticulitis were included in this section.

#### Infectious causes

Cows positive for infectious diseases included in an eradication program such as neosporosis, bovine viral diarrhoea and paratuberculosis.

#### Unknown causes

Unknown cause of death and lack of data on the cow elimination report.

### Collection of data

Clinical, reproduction, production and management data were recorded using specific software (Afifarm, Afimilk, Kibbutz Afikim, 1514800, Israel). The following individual data of the eliminated cows were recorded from the herd database: identification of farm, identification of the cow, date of birth, date of artificial insemination, bull, pregnancy diagnosis, disease diagnosed (date), clinical signs, analytical data, treatment (date and drug), change of pen, preventive hoof trimming, vaccination (date and product), date of calving, date of dry-off, date of death, destiny (slaughtered or dead at farm), cause of elimination, age at death (DL) and number of lactations at elimination (NL). Moreover, individual production data for each cow under study were life milk yield (LMY) (milk produced throughout the cow’s life), total days in milk (tDIM), litres per day of productive life (LPL) (LPL was calculated as LMY/tDIM) and litres per day of life (LDL) (LDL was calculated as LMY/DL).

### Statistical analysis

All statistical analyses were carried out using SAS V.9.1.3 (SAS institute Inc, Cary, NC, USA). For all analyses, the individual cow was used as the study unit. The significance level was set at 0.05. The variables included in the statistical analyses were classified as nominal (farm, destiny and cause of elimination), ordinal (NL and period of the year) or continuous: DL, LMY, tDIM, LPL and LDL. All the cows with five or more lactations were grouped to carry out the statistical analysis. Shapiro–Wilk’s and Levene tests were used to evaluate the normality of the distribution of the continuous variables and the homogeneity of variances, respectively.

Descriptive statistics were performed for all the variables by farm and as an average of the five farms. For clarity in the figures, the causes of elimination have been grouped into “major causes” (causes of elimination with a percentage higher than 15%), “intermediate cause” (causes of elimination with values between 8 and 15%) and “minor causes” (elimination causes with values lower than 8%). Contingency tables (Chi square or Fisher exact tests) were used when the association between nominal and ordinal variables was assessed. To study the association between nominal or ordinal variables with the continuous non-normally distributed variables, the Wilcoxon test (with the Mann–Whitney U test to compare each pair of values) was used. To analyse the association between continuous normally distributed variables and nominal or ordinal variables, an ANOVA test (with Student’s t-test to compare each pair of values) was used.

## Results

### Productive data of cows eliminated

**Fig. 1:**
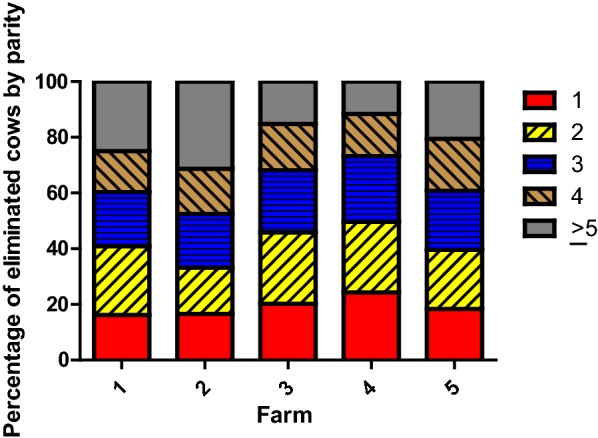
Percentage of eliminated cows by parity and farm

A total of 4811 cows from five dairy farms were either sent to slaughter or died on the farm from 2006 to 2016. On average, eliminated cows had a DL of 1939 ± 11 days, the NL was 3 ± 0.1, the LMY was 31,120 ± 303 l, the tDIM was 984 ± 9 days, the LPL was 29.9 ± 0.2 l and the LDL was 14.4 ± 0.1 l. Significant differences (P < 0.05) were observed between farms for most of the studied parameters with the exception of farms 1 and 5 (Table 2, Additional file 1: Table S1 and Additional file 2: Table S2). Annual herd turnover rate ranged from 25 to 35% on the five farms and the overall percentage of eliminated cows during the first, second, third, fourth and after the fourth parity was 20.4, 22.9, 21.7, 16.2 and 18.8%, respectively. These percentages in eliminated cows considering number of parity were significantly different (P < 0.05) between farms (Fig. 1). Finally, production parameters in slaughtered compared to dead cows were significantly different (P < 0.05): NL (3 ± 0.1 vs 3.2 ± 0.1), DL (1958 ± 12 vs 1861 ± 23 days), tDIM (1003 ± 10 vs 902 ± 19 days), LMY (31,426 ± 338 vs 29,852 ± 685 l) and LPL (29.8 ± 0.1 vs 30.8 ± 03 l) (Table 2).

**Table 2:** Average (± SEM) and confidence intervals (95%) for production parameters of eliminated cows (slaughtered and dead) over 11 years (2006–2016) in five farms

Farm	1	2	3	4	5	Average		
						Eliminated cows	Slaughtered cows	Dead cows
Number of milking cows (n)	140 ± 4	340 ± 9	600 ± 15	420 ± 8	181 ± 6	NA	NA	NA
Number of daily milkings	2	3	3	3	2	NA	NA	NA
NL	3.4 ± 0.1^a^	3.6 ± 0.1^b^	2.9 ± 0.1^c^	2.7 ± 0.1^d^	3.2 ± 0.1^a^	3 ± 0.1	3 ± 0.1^A^	3.2 ± 0.1^B^
	(3.1–3.6)	(3.5–3.7)	(2.8–3.0)	(2.6–2.8)	(3.1–3.3)	(3.0–3.1)	(2.9–3.1)	(3.1–3.3)
DL (days)	2092 ± 45^a^	2171 ± 26^b^	1943 ± 23^c^	1775 ± 15^d^	1935 ± 26^c,e^	1939 ± 11	1958 ± 12^A^	1861 ± 23^B^
	(2004.3–2181.1)	(2120.9–2221.3)	(1897.7–1989.1)	(1745.0–1804.1)	(1884.9–1985.3)	(1913.3–1960.7)	(1935–1981)	(1815–1906)
tDIM (days)	1127 ± 38^a^	1203 ± 21^b^	948 ± 18^c^	842 ± 12^d^	1002 ± 22^e^	984 ± 9	1003 ± 10^A^	902 ± 19^B^
	(1054.6–1202.3)	(1160.8–1245.3)	(912.7–982.6)	(819.5–867.9)	(980.1–1031.5)	(967.8–1001.9)	(984–1022)	(865–939)
LMY (litres)	38,607 ± 1353^a,d^	39,001 ± 765^a^	31,183 ± 629^b^	23,897 ± 374^c^	34,134 ± 787^d^	31,120 ± 303	31,426 ± 338^A^	29,852 ± 685^B^
	(35,467.3–40,782.6)	(37,499.9–40,503.8)	(29,949.6–32,417.3)	(23,156.3–24,623.5)	(32,589.8–35,678.3)	(30,527.4–31,716.2)	(30,763–32,089)	(28,507–31,196)
LPL (litres)	32.4 ± 0.4^a^	30.8 ± 0.2^b^	31.5 ± 0.2^c^	27.0 ± 0.2^d^	32.7 ± 0.2^a^	29.9 ± 0.2	29.8 ± 0.1^A^	30.8 ± 03^B^
	(31.7–33.1)	(30.3–31.2)	(31.2–31.9)	(26.7–27.3)	(32.1–33.1)	(29.8–30.2)	(29.6–30)	(30.3–31.3)
LDL (litres)	16.6 ± 0.4^a^	16.2 ± 0.2^a^	14.6 ± 0.2^b^	12.2 ± 0.1^c^	15.9 ± 0.2^a^	14.4 ± 0.1	14.4 ± 0.1^A^	14.2 ± 0.2^A^
	(16.0–17.5)	(15.8–16.5)	(14.3–15.0)	(11.9–12.4)	(15.6–16.4)	(14.2–14.6)	(14.3–14.6)	(13.7–14.6)

NL, DL, LMY, tDIM, LPL and LDL is number of lactations, days of life, life milk yield, total days in milk, litres per day of productive life and litres per day of life, respectively

*NA* non-applicable

^a–e^ Means within a row with different superscripts differ between farms (*P *< 0.05)

^A, B^Means within a row with different capital superscripts differ between slaughtered and dead cows (*P *< 0.05)

### Destiny and cause of elimination

During the period under study, 80.6% of the eliminated cows were slaughtered and 19.4% died on the farm. Cows were sent to slaughter every 20 days on average and carcasses of dead cows were removed within a mandatory period of 48 h. Significant differences (P < 0.05) were observed between farms such that the highest percentage of dead cows was observed on farms 2 and 5 (20.9 and 25.4%, respectively), whereas the lowest percentage was observed on farm 1 (12.5%). Reproductive disorders (30.2%), production (23.4%) and mammary gland disorders (13.5%) were the three main causes of cow elimination representing 67% of all the events. Secondly, accidents (7.7%), metabolic or digestive disorders (7.2%) and infectious diseases (6.5%) represented 21.4% of the cow eliminations. Thirdly, locomotor disorders (3.6%), dystocia or obstetrics (2.4%) and respiratory causes (0.7%) were a minor group representing 6.7% of all the events. Finally, the cause of elimination was unknown in 4.7% of the cases. The percentage for each cause of elimination and for slaughtered and dead cows by farm is shown in Table 3. Reproductive disorders were the major cause for elimination on farms 1, 2, 3 and 5, whereas the major cause for elimination on farm 4 was productive reasons. Significant differences (P < 0.05) were observed between farms regarding the reason for cow elimination. The causes of elimination were significantly different (P < 0.05) between slaughtered and dead cows. In particular, reproductive disorders, production causes and infectious causes were significantly more frequent (P < 0.05) in slaughtered than in dead cows whereas metabolic/digestive, dystocia/obstetrics, accident, respiratory and unknown causes, were significantly less frequent (P < 0.05) in slaughtered than in dead cows.

**Table 3:** Description of the cause of elimination for cows in the eliminated, slaughtered and dead cows in five farms over 11 years (2006–2016)

Farm	1	2	3	4	5	Average		
						Eliminated cows	Slaughtered cows	Dead cows
Herd turnover (%)	26	25	27	30	35	28.1 (27.0–29.2)	NA	NA
Slaughter (%)	87.5	79.1	81.1	82.2	74.6	80.6 (80.3–82.5)	NA	NA
Dead on the farm (%)	12.5	20.9	18.9	17.8	25.4	19.4 (17.5–19.7)	NA	NA
*Reason for elimination*								
Reproduction (%)	32.8	27.4	40.4	23.7	34.3	30.2 (28.9–31.5)	37.3 (35.8–38.8)	0.7 (0.3–1.5)
Mammary gland (%)	17.7	22.2	10.6	5.7	22.4	13.5 (12.6–14.5)	13.9 (12.9–15.1)	11.6 (9.7–13.8)
Production (%)	14.5	22.3	16.5	31.8	18.0	23.4 (22.2–24.6)	28.9 (27.6.30.4)	0 NA
Locomotor (%)	5.2	4.2	4.3	2.5	3.7	3.6 (3.1–4.2)	3.6 (3–4.2)	3.5 (2.5–4.9)
Metabolic/digestive (%)	13.4	7.8	11.6	2.5	8.6	7.2 (6.5–7.9)	4.1 (3.5–4.7)	20.1 (17.7–22.8)
Respiratory (%)	2.6	0.8	0.7	0.0	1.1	0.7 (0.6–0.8)	0.3 (0.1–0.5)	2.1 (1.3–3.3)
Dystocia/obstetrics (%)	2.0	3.5	2.9	1.5	2.7	2.4 (2.0–2.9)	0.4 (0.2–0.7)	10.7 (8.9–12.8)
Accident (%)	9.3	2.2	6.6	11.2	8.1	7.7 (7.0–8.6)	1.6 (1.3–2)	33.3 (30.3–36.3)
Infectious disease (%)	1.2	3.2	2.4	14.4	0.3	6.5 (5.8–7.3)	6.9 (6.2–7.8)	4.7 (3.5–6.3)
Unknown (%)	1.2	6.2	4.1	6.6	0.7	4.7 (4.2–5.4)	2.7 (2.2–3.3)	13.2 (11.2–15.6)

Confidence intervals (95%) between brackets at the average column

**Fig. 2:**
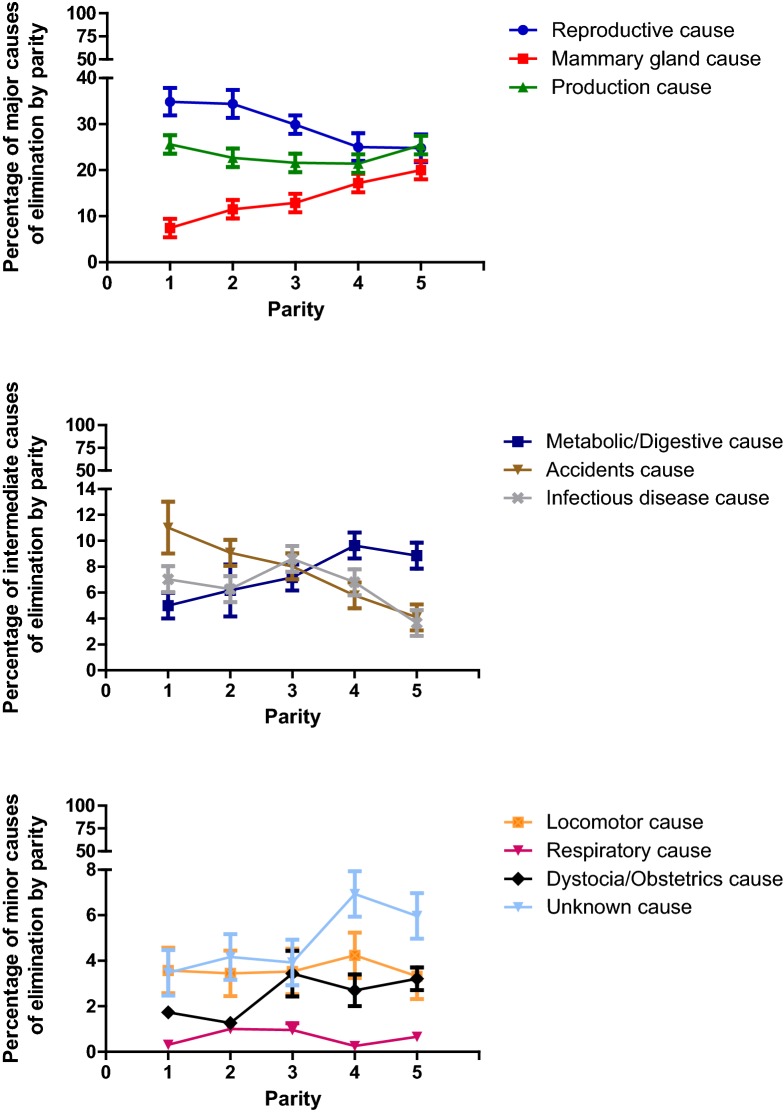
Percentage (with 95% confidence interval) of major, intermediate and minor causes of elimination by cow parity. In the figure, parity equal to or higher than five is represented by five. The causes of elimination have been grouped into “major causes” (causes of elimination with a percentage higher than 15%), “intermediate cause” (causes of elimination with values between 8 and 15%) and “minor causes” (elimination causes with values lower than 8%)

The cause for elimination differed significantly (P < 0.05) depending on the parity of the cow (Fig. 2a–c) but the pattern varied depending on the cause. Reproductive and production causes were the most frequent causes of elimination in cows of all parities. Reproductive cause progressively and significantly (P < 0.05) decreased in importance, especially after more than three lactations. Productive cause also decreased significantly (P < 0.05) in importance between the first and fourth parities, but increased in cows with five or more parities at the same level of first lactation cows. Accidents were the third main cause of elimination for cows of first and second lactation, but progressively and significantly (P < 0.05) decreased in importance. The percentage of eliminated cows due to mammary gland disease, metabolic diseases and unknown causes increased significantly (P < 0.05) as the cows got older. Dystocia showed a significant increase (P < 0.05) in importance between the second and third lactations. Infectious diseases were more important in third lactation cows compared to younger cows and showed a decreasing trend in cows eliminated with four or more lactations. Finally, the percentage of elimination due to locomotor and respiratory causes did not show any statistically significant changes (P > 0.05) during the cows’ lifespan.

**Fig. 3:**
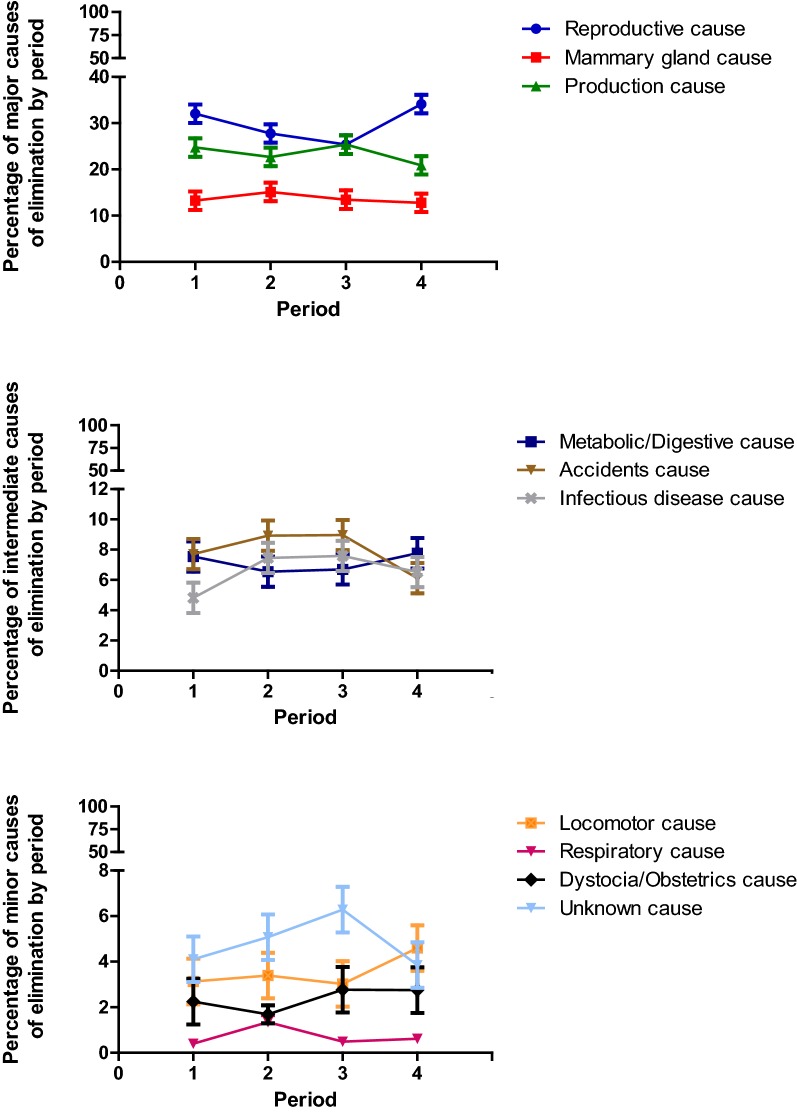
Percentage (with 95% confidence interval) of major, intermediate and minor causes of elimination by period of year. The year was divided into four periods: 1 (1st January–31st March), 2 (1st April–30th June), 3 (1st July–30th September) and 4 (1st October–31st December). The causes of elimination have been grouped into “major causes” (causes of elimination with a percentage higher than 15%), “intermediate cause” (causes of elimination with values between 8 and 15%) and “minor causes” (elimination causes with values lower than 8%)

For all farms, the percentage of cows that died on the farm was significantly (P < 0.05) affected by period. Proportion of dead cows was significantly higher during period 2 (22.4%) and 3 (22.4%) when compared to period 1 (17.9%) and 4 (16.4%). The causes of elimination were also significantly affected by the time of year but the pattern was different taking into account the different causes (Fig. 3a–c). Reproductive and metabolic/digestive causes showed the highest values during periods 1 and 4 that were significantly higher (P < 0.05) than for period 2 and 3, whereas accidents and infectious diseases showed the highest values during periods 2 and 3 that were significantly higher (P < 0.05) than for periods 1 and 4. On the other hand, elimination due to unknown and locomotor causes peaked at periods 3 and 4, respectively without showing significant differences (P > 0.05) with the other periods. The remaining causes of elimination did not show significant variation throughout the year.

## Discussion

This study used data from five dairy herds in Spain from 2006 to 2016 that are typical of the intensive milk production sector in southern Europe, but also in other countries and areas of the world. Defining culling criteria and a cause of mortality can be biased depending on how data are collected, who is responsible for the data collection and on the data analysis system [1]. All data analysed in the study were collected using the same software system and the same team of veterinarians. Use of the same criteria and analysis system is necessary to advice farmers and benchmark different farms. This point is critical because the reason for culling can be biased depending on how data are collected, who is responsible for this data collection and the data analysis system [17]. Despite similarities in management, nutrition, collection and data analysis, veterinarian diagnosis and treatment of diseases, key points of herd performance such as age at death, mortality and culling reasons, tDIM and LMY differed significantly between herds. In this study, these differences could be more related to intrinsic factors of each farm such as care of animals by workers, differences between farmer’s criteria when deciding to treat a sick cow or euthanize it, hygiene and design of facilities than number of milking cows or milk yield. Climate conditions may also have an effect on mortality and cause of death. We suggest that analysing between-herd differences and benchmarking farms is useful to improve the day-by-day management and animal care on the farms.

The DL and LMY of eliminated cows in our study was similar to the value reported in a study from the Netherlands [19]. On the other hand, tDIM was lower than values described by others [20, 21]. Finally NL (3.0 ± 0.1) was lower than the value described by Africor (3.3 ± 0.1) [22] but higher than a Swedish study with multiple dairy breeds (2.3 ± 1.4) [9]. Thus, there is great variability in the productive parameters of eliminated cows published in the literature and comparison to other studies is difficult given the different methods of collection of production data. Some of these studies analyse data based on information provided by official control agencies that estimate production. This makes it imperative to obtain data for a particular geographical area and for particular production characteristics in order to define benchmarking strategies.

In this study, annual average herd turnover rate on the farms ranged between 25 and 35%. The terms used in the literature to define the causes for the elimination of cows, due to either culling or mortality, and whether this elimination is voluntary and planned or involuntary and unplanned, can be confusing. In any case, the percentage of turnover rates ranges from 21.3% in Ireland [23] and up to 40% in Australia [24], 25.4% in the Netherlands [19], 26% in northwest Spain [7], 22.6 and 30% in the UK [25, 26], between 28.2 and 33.5% in Canada [27] and 36% in the USA [28]). In contrast, between-herd differences for herd turnover rates are 10% in this research study, whereas, in other studies in Europe between-herd differences where 50.3% in the Netherlands (herd turnover range 6.2–56.5%) and 24% in the UK (range 11.5–35.5%). These two studies included a greater number of farms, which may have had different production and management systems [29], which could explain these differences. On the other hand, results from the current report agree with those from a study conducted in Poland, with a culling rate between 20 and 35% [30] and rates of 28.1–35.2% reported in another study carried out in the USA with farms whose size was similar to those included in our study [4]. In our study, turnover rates for second and third parity cows (22.9 and 21.7% respectively) were significantly higher than first, fourth and after fourth parity cows (20.4, 16.2 and 18.8%, respectively). Farmers and technicians of the included farms are aware that a cow needs more than one parity to achieve economic amortization. This reference value on economic amortization is based on economic data from the farms under study and is supported by recent research [31]. It could be a reasonable explanation why farmers increase the culling rate during the second and third parity. After the fourth parity, turnover rate decreases probably because cows have demonstrated good production and reproductive performance [14].

In our study, 19.4% of the eliminated cows died on the farm. This result is similar to that reported by other studies with the proportion of dead cows between 17.2 and 20.6% in northwestern Spain and USA [7, 10] but higher than 10.6 and 14.8% reported previously for USA [28, 32]. The proportion of dead cows may be a consequence of socio-psychological differences between countries and regions in terms of practices around euthanasia and death [33]. We suggest that farmers in our study area were more likely to treat sick animals and keep them on the farm than they are in other countries, which would explain the differences in proportion of cows dying on the farms compared to other studies. Another factor that may affect the proportion of dead cows could be the intensity of the heat stress season. It has been shown that this period is a risk factor in significantly increasing mortality on farms and the proportion of dead cows could be extremely high on farms without optimal anti-heat stress systems [14, 15, 34]. Other factors to explain the higher levels of dead cows in this study compared to others [28, 32], could be injured animals due to accidents, mainly related to slippery floors. Additionally, the transport of sick or injured cows to slaughter is strictly regulated and farmers can be penalized with large fines. Thus, they may prefer to have a problematic animal die on the farm instead of sending it to the slaughterhouse. Overall, between-herd differences were observed in this parameter. The percentage of dead cows could be a good parameter for benchmarking farms, since many factors are at play including building design, management, disease prevention and elimination policy.

Regarding the overall data analysis, the most important cause of elimination was reproductive issues followed by low milk production and mammary gland disease. The order of the causes of elimination agreed with other studies, but the proportions for each reason were different between studies. Thus, the percentage of elimination due to reproductive causes was higher (30.2%) than the value described in the USA (17%) [10], Canada (18%) [27], Sweden (24.8%) [35] and north-west Spain (24.7%) [7]. Low milk production was the second most important cause of elimination (23.4%) in our study and it was higher than the value found for USA (12.1%) [10], Canada (6–7%) [27] and Sweden (6.2%) [35]. Mammary gland disease was the third main cause of removal (13.5%), and this percentage was lower than the value reported in Sweden (22.5%) [35] and Spain (15.6%) [7] but similar to the values reported in the USA (12.1%) [14], and Canada (11.3–12.5%) [27]. The next most important causes of elimination were accidents and metabolic and digestive disorders, which also showed a high variability between studies. The percentage of eliminated cows due to accidents on the farm in the current study (7.7%) was higher than the value found for Canada (3.8%) [27], but it was lower than the values reported in Denmark (5–19%) [18]. Finally, the percentage of eliminated cows due to metabolic and digestive diseases (7.2%) was higher than the values reported in Sweden (2.2%) [35].

Elimination of animals positive for infectious diseases was responsible for 6.5% of eliminated cows. Other studies report information on infectious diseases as a cause for elimination, including bovine leucosis or paratuberculosis, but comparisons cannot be carried out due to different ways of collecting information. The percentage of eliminated cows due to foot and leg problems (3.6%) was lower than the value described in USA (8.1%) [10], Spain (8.0%) [7], Canada (7.2–7.8%) [27] and Sweden (6.6%) [35]. Problems at calving were responsible for 2.4% of eliminations. This percentage was higher than the report (0.6%) from Canada [27] but lower than a study that reviewed mortality data worldwide (2.0–46.0%) [18]. A lack of information regarding the reasons for elimination was found in 4.7% of dead animals. This cause of elimination defined as “unknown” is also observed in other studies at similar proportions (4–5%) [10, 36]. Some studies and data centres show a higher proportion (17–20%) of unknown causes of elimination [27, 37]. When a comparison of causes of elimination was carried out between herds, differences were observed suggesting that the analysis of the cause of elimination can also be a proper indicator for benchmarking and improving production systems of farms [29, 38]. Causes of elimination also differed significantly in relation to parity and in a logical pattern. For instance, the importance of mammary gland and metabolic diseases as causes of elimination increased with age in our study. As previously reported by others, the risk of mastitis increases significantly as the number of lactations increases compared to first lactation cows (for second, third and fourth or more lactation cows respectively: RR = 1.57/2.41/3.40) [39]. In the case of metabolic diseases, cows with 3 or more parities have an increased relative risk of subclinical hypocalcaemia (RR = 1.7; 95% CI 1.2–2.3) [40] and ketosis (RR = 1.64; 95% CI 1.59–1.69) when compared to second lactation cows [41]. A comparison of the cause of cow elimination should be carried out carefully among different countries but should also be detailed between farms with very similar characteristics to compare them and identify problems of animal welfare and management, with the objective of providing specific and personalised solutions.

The season of the year significantly affected mortality, increasing during the hottest seasons as reported in other studies. A one unit increase in Heat Stress Index results in a 3% increase in the on-farm mortality rate (RR = 1.03; 95% CI 1.03–1.04) [15] whereas one study reported an increase of 4.7% in mortality associated with a 1 °C increase in Heat Stress Index in Italy [42]. Another study carried out in Belgium showed significant heat-related increases in dairy cattle mortality, (RR = 1.09; 95% CI 1.02–1.17, for moderate heat and RR = 1.26; 95% CI 1.08–1.48, for extreme heat) [34]. These results agree with a Swedish study where the risk of mortality increased during the hot season versus autumn and winter [38]. Differences were also observed between herds, since farms without anti-heat stress systems had higher mortality rates during the warm season. This is of particular interest when proposing investments to mitigate heat stress on farms located in areas or countries where climate change may have an impact. These farms should adapt their production system to the changing climate conditions. Season also significantly affects the cause for removing a cow. As an example, reproductive disorders and metabolic/digestive diseases as causes of elimination increased during period 4 (Sep–Dec). A reasonable explanation could be that these cows suffer from heat stress during the dry off period and suffered from a negative energy balance before parturition [6, 43].

## Conclusions

This study shows that the causes of elimination can be significantly different between dairy farms even though they have similar productive systems, similar health status, close veterinary diagnosis and herd software data compilation. These differences could be due mainly to the farmer’s personality, differences in the cow management and facility design. Applying benchmarking strategies based on data from eliminated cows can be very useful to demonstrate that the elimination plan is not suitable, the management of the animals may be improved or that poorly designed farm facilities may have a negative impact on the efficiency of the farm. In summary, the analysis of the causes of cow elimination can be of great technical interest for the farmer but the same classification of causes and veterinary diagnosis must be used in dairy farms to draw comparable conclusions.

## Additional files

**Additional file 1: Table S1.** Distribution of the number of milking cows, number of lactations (NL) and days of life (DL) of eliminated cows by farm over 11 years (2006–2016).

**Additional file 2: Table S2.** Distribution of the total days in milk (tDIM), life milk yield (LMY), litres per day of productive life (LPL) and litres per day of life (LDL) of eliminated cows by farm over 11 years (2006–2016).

## Abbreviations

DF: dead on the farm
DIM: days in milk
DL: age at death
DS: dead due to slaughter
LDL: litres per day of life
LMY: life milk yield
LPL: litres per day of productive life
NL: number of lactations at elimination
tDIM: total days in milk
