# Supplementary material for: Descriptive study for culling and mortality in five high-producing Spanish dairy cattle farms (2006–2016)
Source: Acta Vet Scand. 2018 Jul 28;60:45. doi: 10.1186/s13028-018-0399-z (PMC6064081; doi:10.1186/s13028-018-0399-z)
Supplement: Supplementary file 1 — Additional file 1: Table S1. Distribution of the number of milking cows, number of lactations (NL) and days of life (DL) of eliminated cows by farm over 11 years (2006–2016). [file 13028_2018_399_MOESM1_ESM.doc]

Additional file 1. Distribution of the number of milking cows, number of lactations (NL) and days of life (DL) of eliminated cows by farm over eleven years (2006-2016).

|  | Number of milking cows (n) | | | NL | | | DL(days) | | |
| --- | --- | --- | --- | --- | --- | --- | --- | --- | --- |
| Farm | Avg. | R | CV (%) | Avg. | R | CV (%) | Avg. | R | CV (%) |
| 1 | 140 | 8 | 1.7 | 3.4 | 9 | 57.2 | 2092 | 3196 | 61.8 |
| 2 | 340 | 17 | 1.4 | 3.6 | 10 | 54.2 | 2171 | 3552 | 57.1 |
| 3 | 600 | 21 | 1.2 | 2.9 | 8 | 53.7 | 1943 | 3188 | 59.7 |
| 4 | 420 | 10 | 0.1 | 2.7 | 10 | 53.9 | 1775 | 4354 | 61.1 |
| 5 | 181 | 7 | 1.5 | 3.2 | 8 | 53.6 | 1935 | 2970 | 57.1 |

NA Non-applicable

CV Coefficient of Variation

Avg Average

R Range (Maximum minus minimum)
