# Supplementary material for: Descriptive study for culling and mortality in five high-producing Spanish dairy cattle farms (2006–2016)
Source: Acta Vet Scand. 2018 Jul 28;60:45. doi: 10.1186/s13028-018-0399-z (PMC6064081; doi:10.1186/s13028-018-0399-z)
Supplement: Supplementary file 2 — Additional file 2: Table S2. Distribution of the total days in milk (tDIM), life milk yield (LMY), litres per day of productive life (LPL) and litres per day of life (LDL) of eliminated cows by farm over 11 years (2006–2016). [file 13028_2018_399_MOESM2_ESM.doc]

Additional file 2. Distribution of the total days in milk (tDIM), life milk yield (LMY), litres per day of productive life (LPL) and litres per day of life (LDL) of eliminated cows by farm over eleven years (2006-2016).

|  | tDIM (days) | | | LMY (litres) | | | LPL (litres) | | | LDL (litres) | | |
| --- | --- | --- | --- | --- | --- | --- | --- | --- | --- | --- | --- | --- |
| Farm | Avg. | R | CV (%) | Avg. | R | CV (%) | Avg. | R | CV | Avg. | R | CV (%) |
| 1 | 1127 | 3816 | 39.9 | 38607 | 113115 | 65.8 | 32.4 | 41.7 | 20.7 | 16.6 | 40.4 | 42.0 |
| 2 | 1203 | 4194 | 37.6 | 39001 | 136792 | 62.7 | 30.8 | 44.9 | 23.0 | 16.2 | 29.8 | 37.5 |
| 3 | 948 | 4523 | 38.0 | 31183 | 118592 | 64.0 | 31.5 | 44.9 | 17.6 | 14.6 | 28.7 | 37.9 |
| 4 | 842 | 4474 | 35.5 | 23897 | 89750 | 65.4 | 27.0 | 45.9 | 24.3 | 12.2 | 26.9 | 43.7 |
| 5 | 1022 | 3606 | 34.9 | 34134 | 111889 | 60.8 | 32.7 | 46.1 | 17.4 | 15.9 | 29.3 | 36.2 |

NA Non-applicable

CV Coefficient of Variation

Avg Average

R Range (Maximum minus minimum)
